# Vascularization, Oxygenation, and the Effect of Sunitinib Treatment in Pancreatic Ductal Adenocarcinoma Xenografts

**DOI:** 10.3389/fonc.2019.00845

**Published:** 2019-08-29

**Authors:** Jon-Vidar Gaustad, Trude G. Simonsen, Catherine S. Wegner, Einar K. Rofstad

**Affiliations:** Group of Radiation Biology and Tumor Physiology, Department of Radiation Biology, Institute for Cancer Research, Oslo University Hospital, Oslo, Norway

**Keywords:** pancreatic ductal adenocarcinoma, sunitinib, vascular normalization, hypoxia, dorsal window chamber

## Abstract

The purpose of the study was to investigate vascularization, oxygenation, and the effect of sunitinib treatment in pancreatic ductal adenocarcinoma (PDAC). BxPC-3 and Capan-2 xenografts grown in dorsal window chambers were used as preclinical models of human PDAC. Tumor angiogenesis and the morphology and function of tumor vascular networks were studied by intravital microscopy, and tumor hypoxia was assessed by immunohistochemistry. The PDAC models differed in vessel distribution and oxygenation, and the differences were induced by the initial tumor angiogenesis. In both models, sunitinib treatment reduced intratumor and peritumor vessel densities by selectively removing small-diameter vessels. Sunitinb treatment resulted in a general decrease in vessel density and scattered hypoxia in BxPC-3 tumors, and depleted most vessels and induced massive hypoxia in central parts of Capan-2 tumors. The study demonstrates that PDAC xenografts can differ in vascularization, and the differences can impact oxygenation and effects of treatment. Neoadjuvant sunitinib treatment is inappropriate in combination with conventional therapy for human PDACs resembling the PDAC xenografts used here, because sunitinib-induced hypoxia can impair the effect of most conventional therapies.

## Introduction

Pancreatic ductal adenocarcinoma (PDAC) is a highly aggressive disease associated with poor prognosis and a 5-year survival rate of 5–7% (1, 2). The only curative treatment option for PDAC patients is surgical resection, but most cases are too advanced for surgery at the time of diagnosis. The majority of PDAC patients receive chemotherapy, alone or in combination with radiation therapy, but these treatments only prolong survival marginally (3). The reason for the poor survival rates is not fully understood but treatment resistance caused by the tumor microenvironment is believed to be an important component. Several studies have demonstrated that poor blood supply and severe hypoxia in the primary tumor is associated with poor prognosis for PDAC patients (4–6).

In the 1970s, Folkman demonstrated that tumor growth is angiogenesis-dependent, and suggested that antiangiogenic therapy could suppress tumor growth by starving tumor cells (7). After this pioneering work, several antiangiogenic drugs were developed and tested in clinical trials (8). Most of the drugs targeted the vascular endothelial growth factor (VEGF) pathway, either by blocking VEGF-A with neutralizing antibodies such as avastin, or by blocking the VEGF-receptors with tyrosine kinase inhibitors such as sunitinib (8). Unfortunately, none of the antiangiogenic therapies improved survival for PDAC patients (9). More recently, Jain and others demonstrated that antiangiogenic therapy can be designed to normalize tumor vasculature (10). The vascular normalization strategy involves low doses and short treatment periods, and is intended to sensitize the tumor tissue to conventional therapy. Vascular normalization has been shown to increase tumor blood supply and oxygenation, and to enhance the effect of ionizing radiation, chemotherapy, and immunotherapy in preclinical tumor models (11–14). Clinical trials designed to normalize tumor vasculature have not been reported for PDAC patients, but in a recent review, Li et al. concluded that the vascular normalization strategy has shown great clinical potential and represents a promising future direction of vasculature-targeted treatment in pancreatic cancer (9). However, vascular normalization is debated because some investigators have shown that antiangiogenic drugs fail to normalize the vasculature in some preclinical models (15, 16).

Surgically implanted dorsal window chambers allow high-resolution microscopy imaging of living tissue, and are particularly well-suited to study tumor vasculature and treatment-induced effects on tumor vasculature (17). Despite this, the dorsal window chamber assay has not been used to study effects of antiangiogenic treatment in PDAC xenografts. In the current study, the effect of sunitinib treatment was investigated in two PDAC xenograft models growing in dorsal window chambers. The PDAC models differed in vascularization and oxygenation, and sunitinb treatment reduced vessel densities and induced hypoxia in both models.

## Materials and Methods

### Tumor Models

BxPC-3 and Capan-2 (American Type Culture Collection, VA, USA) human PDAC xenografts grown in dorsal window chambers were used as tumor models. Window chambers were surgically implanted in the dorsal skin fold of adult female BALB/c *nu/nu* mice as reported previously (18). Briefly, the chamber consisted of two parallel frames that sandwiched an extended double layer of skin. Before the chamber was implanted, a circular hole with a diameter of ~6.0 mm was made in one of the skin layers. A plastic window with a diameter of 6.0 mm was attached to the frame on the surgical side with a clip to provide visual access to the fascial side of the opposite skin layer. Tumors were initiated by implanting tumor fragments with a diameter of ~1 mm directly onto the fascial side of the intact skin layer. The tumor fragments were harvested from BxPC-3 or Capan-2 xenografts growing intramuscularly as described previously (19).

### Anesthesia

Window chamber implantation and intravital microscopy examinations were carried out with anesthetized mice. Fentanyl citrate (Janssen Pharmaceutica, Beerse, Belgium), fluanisone (Janssen Pharmaceutica), and midazolam (Hoffmann-La Roche, Basel, Switzerland) were administered intraperitoneally in doses of 0.63, 20, and 10 mg/kg, respectively.

### Sunitinib Treatment

Sunitinb L-malate (LC Laboratories, Woburn, MA, USA) was dissolved as described previously (20). Mice were treated with 40 mg/kg/day sunitinib or vehicle for 4 days, by oral administration.

### Intravital Microscopy

Mice with window chambers were fixed to the microscope stage during intravital microscopy, and the body core temperature was kept at 37–38°C by using a hot-air generator. Imaging was performed by using an inverted fluorescence microscope (IX-71; Olympus, Munich, Germany) and a black and white CCD camera (C9300-024; Hamamatsu Photonics, Hamamatsu, Japan) (20). Tumor vessels perfused with red blood cells were visualized by using a ×4 objective lens, transillumination, and filters for green light resulting in images with a pixel size of 3.7 × 3.7 μm^2^. To study the function of tumor vasculature, first-pass imaging movies were recorded after a 0.2 mL bolus of 50 mg/mL tetramethylrhodamine isothiocyanate-labeled dextran (TRITC; Sigma-Aldrich) with a molecular weight of 155 kDa was injected into the lateral tail vein. First-pass imaging movies were recorded at a frame rate of 22.3 frames per second by using a ×2 objective lens, resulting in a time resolution of 44.8 ms and a pixel size of 7.5 × 7.5 μm^2^. High resolution images of plasma perfused vessels were recorded by using TRITC fluorescence and a ×4 objective lens (pixel size = 3.7 × 3.7 μm^2^).

### Analysis of Vascular Morphology and Function

Vessel length density (i.e., total vessel length per mm^2^ tumor area) and mean vessel diameter were computed from manually produced vascular masks by applying algorithms implemented in MATLAB software (The MathWorks, Natick, MA, USA), as previously described (18, 20). Vessel segment length (i.e., the distance between the branching points along the vessel) was calculated from ~50 randomly selected vessel segments. Blood supply time (BST) images were produced from first-pass imaging movies by assigning a BST value to each pixel of the vascular masks (21). The BST of a pixel was defined as the time difference between the frame showing maximum fluorescence intensity in the pixel and the frame showing maximum fluorescence intensity in the main tumor supplying artery, as described in detail previously (22). Fractional vessel length with only plasma flow (*F*_*PF*_) was quantified by measuring the total vessel length in fluorescence images (*VL*_*F*_) and trans-illumination images (*VL*_*T*_), and was defined as *F*_*PF*_ = (*VL*_*F*_–*VL*_*T*_)/*VL*_*F*_ × 100%.

### Immunohistochemical Detection of Tumor Hypoxia and Microvessels

The tumors were resected immediately after the last intravital microscopy examination and fixed in phosphate-buffered 4% paraformaldehyde. Pimonidazole [1-[(2-hydroxy-3-piperidinyl)-propyl]-2-nitroimidazole], administered as described previously (23), was used as a hypoxia marker and CD31 was used as a marker for endothelial cells. An anti-pimonidazole rabbit polyclonal antibody (Professor James A. Raleigh, University of North Carolina, Chapel Hill, NC, USA) or an anti-CD31 rabbit polyclonal antibody (Abcam, Cambridge, UK) was used as primary antibody. Diaminobenzidine was used as chromogen, and hematoxylin was used for counterstaining. Hypoxic fractions were assessed by image analysis and were defined as the area fraction of the viable tissue showing positive pimonidazole staining. Number of CD31-positive microvessel profiles per mm^2^ of tissue (#/mm^2^) was scored manually and used as parameter for microvascular density (MVD).

### Statistical Analysis

Statistical comparisons of data were carried out by the Student's *t* test when the data complied with the conditions of normality and equal variance. Under other conditions, comparisons were done by non-parametric analysis using the Mann-Whitney rank sum test. The Kolmogorov-Smirnov method was used to test for normality, and the Levene's test was used to test for equal variance. Probability values of *P* < 0.05, determined from two-sided tests, were considered significant. The Pearson product moment correlation test was used to search for correlations between two parameters. The statistical analysis was performed by using the SigmaStat statistical software (SPSS Science, Chicago, IL, USA).

## Results

### BxPC-3 and Capan-2 Tumors Differed in Vascularization and Oxygenation

**Figure 1:**
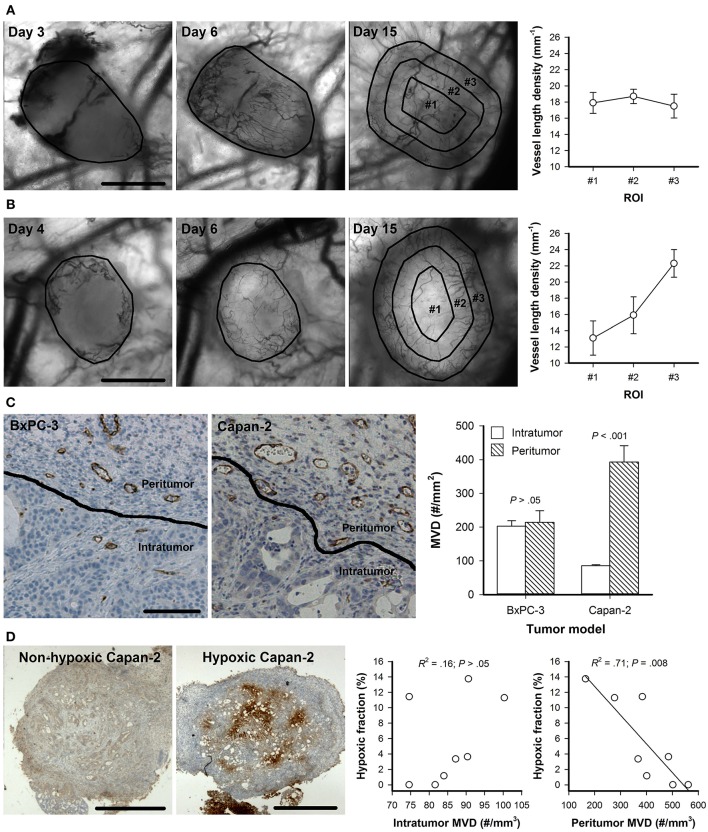
**(A,B)** Intravital microscopy images of a representative BxPC-3 **(A)** and Capan-2 tumor **(B)** recorded 3–4, 6, and 15 days after tumor initiation, and vessel length density in concentric circular regions of interest (ROI) in BxPC-3 and Capan-2 tumors with established vascular networks (day 15). Tumor area and ROI #1-#3 are delineated by black lines in the intravital microscopy images. Symbols: means of 6–8 tumors, error bars: SEM. **(C)** Immunohistochemical preparations stained for microvessels, and intratumor and peritumor microvascular density (MVD) in BxPC-3 and Capan-2 tumors. The images show tumor tissue (intratumor) and the surrounding normal tissue (peritumor) of a representative BxPC-3 and Capan-2 tumor. Columns: means of 6–8 tumors, error bars: SEM. **(D)** Immunohistochemical preparations stained for hypoxia of two Capan-2 tumors with different oxygenation status, and hypoxic fraction vs. intratumor MVD and peritumor MVD in Capan-2 tumors. Symbols: individual tumors, curves: linear regression lines. Scale bars: 1 mm **(A,B,D)** or 100 μm **(C)**.

BxPC-3 and Capan-2 tumor fragments induced strong angiogenic responses in dorsal window chambers as illustrated in Figures 1A,B. In BxPC-3 tumors, novel vessels were observed in central and peripherial parts of the tumors 3–4 days after tumor initiation, and the tumor masses were vascularized by vessels growing from these initial vessels (Figure 1A). In Capan-2 tumors, initial vessels were only observed in peripherial parts of the tumors, and the tumors were vascularized by vessels growing from the tumor periphery (Figure 1B). The established vascular networks had a homogenous vessel distribution in BxPC-3 tumors (Figure 1A, day 15), whereas Capan-2 tumors showed a radial heterogeneity in vessel density, i.e., the vessel length density was low in central tumor regions and increased toward the tumor periphery (Figure 1B, day 15). Abnormal vessels were also observed outside the tumors (peritumor), and immunohistochemical preparations were stained for microvessels to quantify intratumor and peritumor MVD (Figure 1C). Peritumor MVD was higher than intratumor MVD in Capan-2 tumors (*P* < 0.001), whereas BxPC-3 tumors showed similar intratumor and peritumor MVD (*P* > 0.05). BxPC-3 tumors showed higher intratumor MVD than Capan-2 tumors (*P* < 0.001), and Capan-2 tumors showed higher peritumor MVD than BxPC-3 tumors (*P* = 0.011). Immunohistochemical preparations were also stained for hypoxia to quantify hypoxic tumor fractions (Figure 1D). BxPC-3 tumors did not show hypoxic regions suggesting that the intratumor MVD was sufficiently high to supply the tumor tissue with enough oxygen. Six out of 8 Capan-2 tumors showed hypoxic regions, and the hypoxic fraction varied from 1.2 to 13.7%. Significant correlations were not found between intratumor MVD and hypoxic fraction (*P* >*0.0*5; Figure 1D), but were found between peritumor MVD and hypoxic fraction (*P* = 0.008; Figure 1D). These observations suggest that the density of peritumor vessels governed the extent of hypoxia in Capan-2 tumors.

### Sunitinib Treatment Removed Both Intratumor and Peritumor Vessels

**Figure 2:**
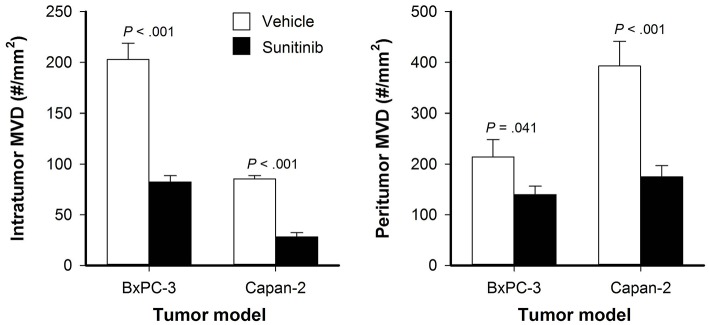
Intratumor and peritumor microvascular density (MVD) in untreated and sunitinib-treated BxPC-3 and Capan-2 tumors. Columns: means of 6–8 tumors, bars: SEM.

BxPC-3 and Capan-2 tumors were divided into size-matched groups to receive sunitinib treatment or vehicle after the tumors had established vascular networks. Sunitinb-treated BxPC-3 and Capan-2 tumors showed lower intratumor and peritumor MVD than untreated tumors, suggesting that the treatment removed both intratumor and peritumor vessels (*P* < 0.05; Figure 2).

### Sunitinib Treatment Selectively Removed Small-Diameter Vessels

**Figure 3:**
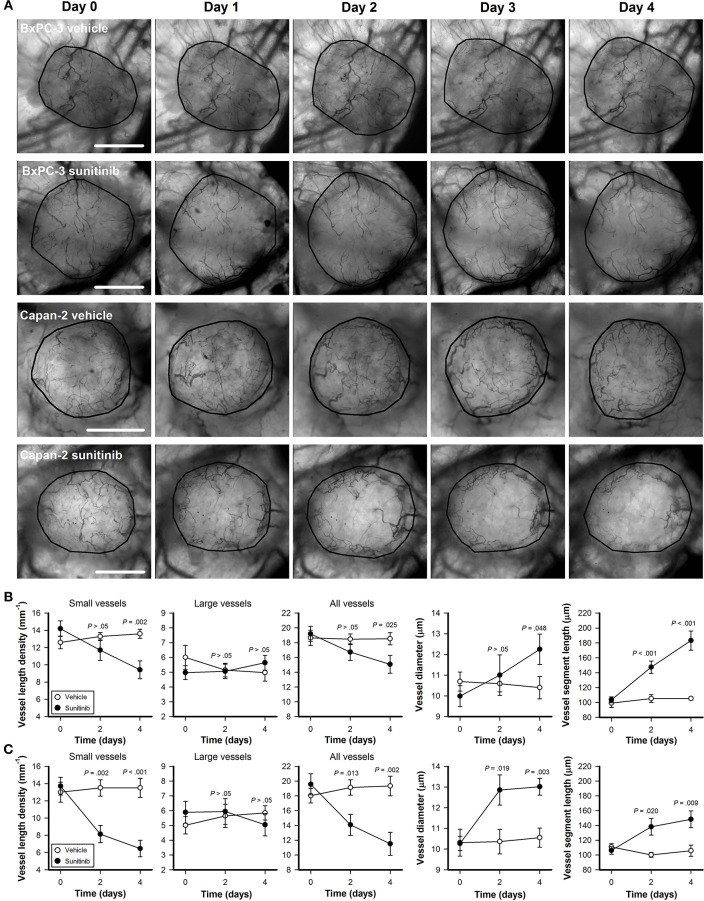
**(A)** Intravital microscopy images of representative untreated and sunitinib-treated BxPC-3 and Capan-2 tumors recorded before (day 0) and during the treatment period (day 1–4). Tumor area is delineated by a black line. Scale bars: 1 mm. **(B,C)** Vessel length density, vessel diameter, and vessel segment length vs. time in untreated and sunitinib-treated BxPC-3 **(B)** and Capan-2 tumors **(C)**. Vessel length density was quantified by including only small-diameter vessels (diameter <10 μm), only large-diameter vessels (diameter >10 μm), or all vessels. Symbols: means of 6–8 tumors, error bars: SEM.

BxPC-3 and Capan-2 tumors were subjected to daily intravital microscopy during the treatment period. Figure 3A shows intravital microscopy images recorded before the treatment started (day 0) and during the treatment period (day 1–4) of representative untreated and sunitinib-treated BxPC-3 and Capan-2 tumors. The images illustrate that sunitinb treatment depleted most vessel in central parts of Capan-2 tumors, and induced a general decrease in vessel density in BxPC-3 tumors. Quantitative studies revealed that sunitinib treatment reduced the density of small-diameter vessels (*P* < 0.01; Figures 3B,C, diameter <10 μm), and did not alter the density of large-diameter vessels (*P* > 0.05; Figures 3B,C, diameter >10 μm). The selective removal of small-diameter vessels reduced overall vessel length density (all vessels) and increased vessel diameter and vessel segment length in sunitinib-treated tumors (*P* < 0.05; Figures 3B,C).

### Sunitinib Treatment Reduced Blood Supply Times

**Figure 4:**
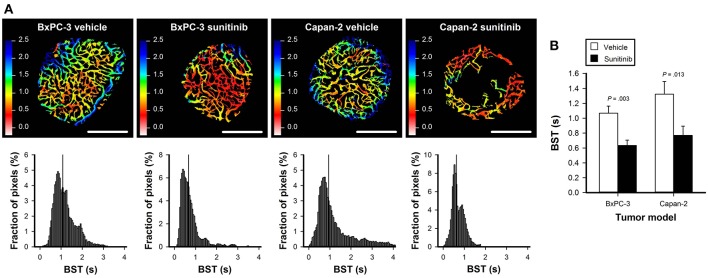
**(A)** Blood supply time (BST) images and the corresponding BST frequency distributions of representative untreated and sunitinib-treated BxPC-3 and Capan-2 tumors. Color bars: BST scale in seconds, scale bars: 1 mm. **(B)** Median BST in untreated and sunitinib-treated BxPC-3 and Capan-2 tumors. Columns: means of 6–8 tumors, error bars: SEM.

To study sunitinib-induced effects on vascular function, first-pass imaging movies were recorded and BST images and BST frequency distributions were produced. Figure 4A shows the BST image and the corresponding BST frequency distribution of representative untreated and sunitinib-treated BxPC-3 and Capan-2 tumors. In both PDAC models, sunitinib-treated tumors showed lower BST values than untreated tumors, implying that vessels with high BST and hence low blood flow velocities were removed by the treatment (BxPC-3: *P* = 0.003, Capan-2: *P* = 0.013; Figure 4B).

### Sunitinib Treatment Removed Vessels With Only Plasma Flow

**Figure 5:**
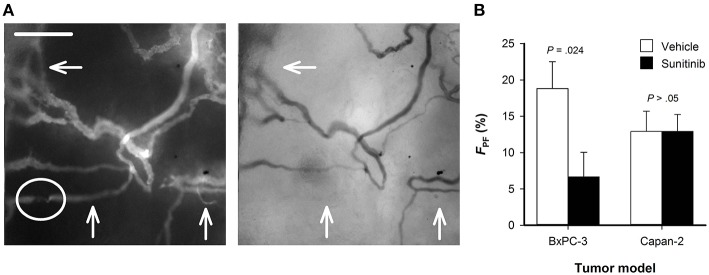
**(A)** Fluorescence and trans-illumination images showing plasma perfused and red blood cells perfused vessels, respectively. Arrows indicate vessels that are clearly visible in the fluorescence image and cannot be detected in the trans-illumination image. These vessels had a low diameter or showed regions that were partly compressed (highlighted with circle) and only carried plasma flow. **(B)** Fractional vessel length with only plasma flow (*F*_*PF*_) in untreated and sunitinib-treated BxPC-3 and Capan-2 tumors. *F*_*PF*_ was defined as *F*_*PF*_ = (*VL*_*F*_–*VL*_*T*_)/*VL*_*F*_ × 100%, where *VL*_*F*_ and *VL*_*T*_ are the total vessel length measured in fluorescence images and trans-illumination images, respectively. Columns: means of 6–8 tumors, error bars: SEM.

High-resolution microscopy images were recorded by using trans-illumination and TRITC fluorescence. The trans-illumination images were recorded with a filter for green light and visualize vessels perfused with red blood cells because these cells absorb green light. TRITC fluorescence images show plasma-perfused vessels regardless of whether the vessels also carry red blood cells, because intravenously administered TRITC-dextran is distributed in the blood plasma. When fluorescence and trans-illumination images of the same field of view were compared, most vessels were clearly visible in both images (Figure 5A). However, some vessels observed in the fluorescence images could not be detected in the trans-illumination images. In Figure 5A, three examples are indicated with arrows. These vessels generally had a low diameter or showed regions that were partly compressed and only carried plasma flow. *F*_PF_ was calculated from the total vessel length measured separately in fluorescence and trans-illumination images, and was lower in sunitinib-treated BxPC-3 tumors than in untreated BxPC-3 tumors (Figure 5B; *P* = 0.024). In Capan-2 tumors, *F*_PF_ was lower and did not differ between sunitinib-treated and untreated tumors (Figure 5B; *P* > 0.05).

### Sunitinib Treatment Induced Tumor Hypoxia and Necrosis

**Figure 6:**
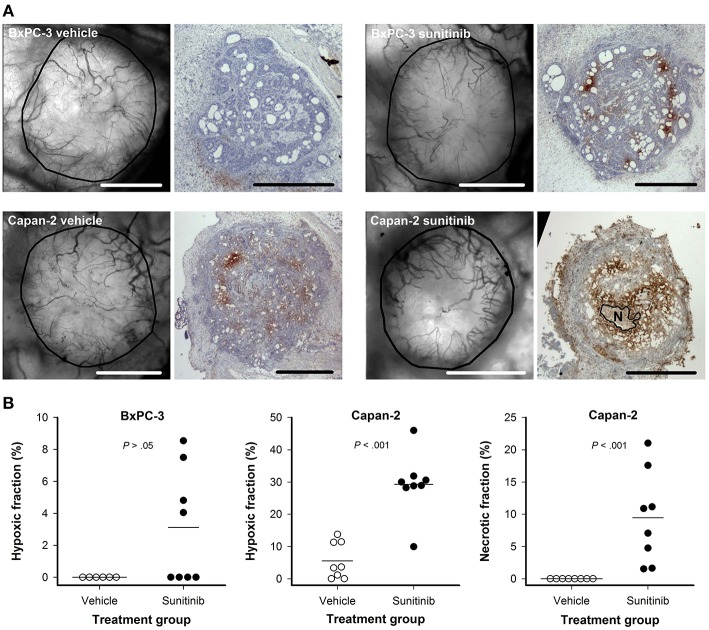
**(A)** Intravital microscopy images and immunohistochemical preparations stained for hypoxia of representative untreated and sunitinib-treated BxPC-3 and Capan-2 tumors. N: central necrosis, scale bars: 1 mm. **(B)** Hypoxic fraction in untreated and sunitinib-treated BxPC-3 and Capan-2 tumors, and necrotic fraction in untreated and sunitinib-treated Capan-2 tumors. Symbols: individual tumors, lines: mean values.

Intravital microscopy images of the tumor vasculature and immunohistochemical preparations stained for hypoxia are shown in Figure 6A. Untreated BxPC-3 tumors did not show hypoxic regions whereas scattered hypoxic regions were found in 4 out of 8 sunitinib-treated BxPC-3 tumors. The sunitinb-induced hypoxic regions generally reflected low overall vessel density, but the difference in hypoxic fraction between untreated and treated BxPC-3 tumors did not reach statistical significance (*P* > 0.05; Figure 6B). Sunitinib-treated Capan-2 tumors showed higher hypoxic fractions than untreated Capan-2 tumors (*P* < 0.001; Figure 6B), and regions with necrotic tissue were found in treated but not in untreated tumors (*P* < 0.001; Figure 6B). The hypoxic and necrotic regions co-localized with avascular regions and were found in central parts of the sunitinib-treated Capan-2 tumors.

## Discussion

Several preclinical models are being used to study PDAC and each model has strengths and limitations (24). In the study reported here, human PDAC xenografts were initiated in dorsal window chambers in immunodeficient mice. The window chamber preparations allowed daily intravital microscopy but also represented a limitation because the subcutaneous microenvironment in immunodeficient mice differs from the microenvironment in human PDAC. However, the range of MVD measured in untreated window chamber tumors (75–320 #/mm^2^) was well within the range of MVD reported in a study of 63 PDAC patients (50–400 #/mm^2^) (25), and the range of hypoxic fractions measured in untreated window chambers tumors (0–14%) was well within the range of hypoxic fractions measured in a study of orthotopic xenograft models derived from 16 PDAC patients (0–20%) (5). Taken together these studies suggest that the PDAC xenograft models used here should be relevant models of human PDAC for studies of vascular development and vascular mechanisms causing hypoxia.

Vessels and hypoxic tissue were visualized by immunohistochemical staining and intravital microscopy. Hypoxic tumor fractions and vessel densities within tumors and in the tissue surrounding tumors were quantified in immunohistochemical preparations, but these techniques required fixation of tissue and could only be performed once. Intravital microsopy allowed quantification of several morphological and functional parameters of tumor vasculature and could be performed multiple times to monitor tumor growth, vascular development, and effects of treatment. The two techniques provided complimentary information, and were combined to search for vascular mechanisms causing hypoxia in both untreated and sunitinib-treated PDAC xenografts. To our knowledge this is the first study evaluating the effect of any antiangiogenic treatment by intravital microscopy in PDAC xenografts.

Two PDAC models were included and the models differed in vascularization and oxygenation. Untreated BxPC-3 tumors showed high intratumor MVD, a homogenous vessel distribution, and no hypoxic regions, implying that these tumors were supplied with sufficient amounts of oxygen. In contrast, established Capan-2 tumors showed low intratumor MVD, a radial heterogeneity in vessel density, and regions with hypoxic tissue before treatment. In addition, an inverse correlation was found between peritumor MVD and hypoxic fraction in Capan-2 tumors, implying that the density of peritumor vessels governed the extent of hypoxia in these tumors, possibly because the intratumor MVD was low.

The tumor-line specific differences in vessel density and distribution were probably caused by differences in the initial tumor angiogenesis. Thus, Capan-2 tumors were vascularized from initial vessels located exclusively in the tumor periphery whereas initial vessels were found in both central and peripherial regions in BxPC-3 tumors. These observations imply that PDACs may exhibit different vascularization patterns, and the vascularization patterns can affect tumor oxygenation and effects of treatment.

Numerous studies have reported the effect of antiangiogenic drugs on intratumor vessels but studies reporting the effect of these drugs on peritumor vessels are sparse. Here we demonstrate that sunitinib treatment reduced both intratumor and peritumor MVD in PDAC xenografts. Moreover, we show that the decrease in vessel density was caused by a selective removal of small-diameter vessels. The latter observation is consistent with several studies demonstrating that antangiogenic agents selectively remove immature blood vessels (12, 15, 26). Small-diameter vessels are expected to have a high geometric resistance to blood flow because the geometric resistance in single vessels is inversely proportional to the vessel diameter in the forth power (27). Accordingly, sunitinib-treated tumors showed lower BST values than untreated tumors implying that the treatment removed vessels with high BST and thus low blood flow velocity.

The removal of intratumor and peritumor vessels decreased oxygen supply and induced hypoxia in both PDAC models. Sunitinib-treated BxPC-3 tumors showed a general reduction in vessel density and scattered hypoxia whereas sunitinib treatment depleted most vessels and induced massive hypoxia and necrosis in central parts of Capan-2 tumors. These differences reflected the tumor-line specific differences in vascularization because untreated BxPC-3 tumors showed high vessel density and a homogenous vessel distribution whereas Capan-2 tumors showed low vessel density in central parts of the tumor before treatment.

The effect of antiangiogenic treatments on tumor oxygenation is controversial. Some investigators have shown that antiangiogenic agents increase tumor oxygenation by normalizing the tumor vasculature, and others have shown that the same agents fail to normalize tumor vasculature and induce hypoxia in other tumor models (12, 14, 15). In tumor models where antiangiogenic treatments increase oxygenation, the treatments have been shown to increase the effect of ionizing radiation and chemotherapy (12–14), but if antiangiogenic treatments induce hypoxia, the treatments are expected to make tumors more resistant to most conventional therapies (16). Experiments combining sunitinib treatment with radiation therapy or chemotherapy were not performed in the current study. However, sunitinib treatment induced hypoxia in both PDAC xenograft models. The study reported here thus argues against neoadjuvant sunitinib treatment in combination with radiation therapy or chemotherapy for PDAC patients, but does not exclude the possibility that sunitinib treatment may inhibit tumor angiogenesis and tumor regrowth if applied after conventional therapies.

Tumor hypoxia has been demonstrated to induce invasive growth and metastatic spread by several mechanisms including the epithelial-mesenchymal transition, increased angiogenesis, altered metabolism, and degradation of the extracellular matrix (28). One could thus speculate whether sunitinib-induced hypoxia may increase aggressiveness in BxPC-3 and Capan-2 xenografts. In accordance with this speculation, accelerated metastasis has been reported by others after antiangiogenic treatment in preclinical tumor models (29, 30).

It has been argued that appropriate timing and low doses of antiangiogenic drugs is required to increase tumor oxygenation because any beneficial effect on vascular function can be balanced by severe vascular depletion after prolonged treatment or if the treatment dose is too high (10, 20, 31). In the current study, the effect of sunitinib treatment was assessed daily by intravital microscopy (day 1–4) and immediately after the treatment period by immunohistochemistry (day 4). These time points correspond well to the time points where improved oxygenation have been reported following avastin (day 1–4) and sunitinib treatment (day 2–4) in other tumor models (12, 32). Moreover, both the same (40 mg/kg) and higher sunitinib doses (50 mg/kg and 100 mg/kg) have been shown to improve vascular function in models of glioblastoma, squamous cell carcinoma, and reneal cell carcinoma (32–34). It is thus unlikely that improved vascular function and oxygen supply could have been observed at other time points or by using lower sunitininb doses in the PDAC models used here.

Vessels that are partly compressed or have a diameter that is small compared to the size of red blood cells can have plasma flow without red blood cell flux. Vessels carrying only plasma flow have been observed in murine tumors and human melanoma xenografts, but have not been reported in PDAC xenografts (35, 36). Moreover, treatment-induced effects on such vessels have not been investigated in any tumor model. In the current study, we demonstrate that both BxPC-3 and Capan-2 tumors have vessels with only plasma flow, and show that sunitinib treatment decreases the fraction of vessels with only plasma flow in BxPC-3 tumors. Vessels with only plasma flow supply little oxygen, but may carry therapeutic molecules. Removal of these vessels is thus not expected to reduce oxygenation but may reduce the uptake of therapeutic drugs.

## Conclusions

Sunitinib treatment selectively removed small-diameter vessels in BxPC-3 and Capan-2 tumors. The treatment resulted in avascular regions and massive hypoxia in Capan-2 tumors, and induced a general reduction in vessel density and scattered hypoxia in BxPC-3 tumors. The different consequences of sunitinib treatment reflected differences in the vascularization pattern of the PDAC models. The study implies that PDACs can differ in vascularization, and the differences can influence tumor oxygenation and treatment effects. Because sunitinib treatment can induce tumor hypoxia and reduce the uptake of therapeutic drugs, neoadjuvant sunitinib treatment may be inappropriate in combination with radiation therapy or chemotherapy for PDAC patients.

## Data Availability

The datasets generated for this study are available on request to the corresponding author.

## Ethics Statement

The animal experiments were approved by the Institutional Committee on Research Animal Care, Department of Comparative Medicine, Oslo University Hospital, Norway and the Norwegian Food Safety Authority (Mattilsynet), Brumunddal, Norway (approved protocol: FOTS #10304), and were performed in accordance with the Interdisciplinary Principles and Guidelines for the Use of Animals in Research, Marketing, and Education (New York Academy of Sciences, New York, NY, USA) and the EU Directive 2010/63/EU for animal experiments.

## Author Contributions

J-VG, TS, CW, and ER conceived and designed the study, analyzed, and interpreted the data. J-VG performed the experiments and wrote the manuscript. All authors read and approved the final manuscript.

### Conflict of Interest Statement

The authors declare that the research was conducted in the absence of any commercial or financial relationships that could be construed as a potential conflict of interest.
